# Prevalence, Severity, and Neuropsychiatric Correlates of Internet Gaming Disorder Among Saudi University Student Gamers: An Epidemiological Cross-Sectional Study

**DOI:** 10.3390/jcm15166374

**Published:** 2026-08-18

**Authors:** Abdullah Alharbi, Faihan F. Alshaibany, Majed M. Aljabri, Bandar S. Alharbi, Alya Alghamdi, Bader M. Almutairy, Waleed M. Alshehri, Norah M. Alyahya, Abdulaziz M. Alodhailah

**Affiliations:** 1 Community and Psychiatric Mental Health Nursing Department, College of Nursing, King Saud University, Riyadh 11451, Saudi Arabia; 2 Department of Nursing Administration and Education, College of Nursing, King Saud University, Riyadh 11451, Saudi Arabia; 3 Department of Medical-Surgical Nursing, College of Nursing, King Saud University, Riyadh 11451, Saudi Arabia

**Keywords:** Internet Gaming Disorder, prevalence, epidemiology, impulsivity, psychological resilience, ROC analysis

## Abstract

**Background**: Despite the WHO’s recognition of Gaming Disorder in the ICD-11, epidemiological data on its prevalence and neuropsychiatric correlates among Saudi Arabian university students remain limited. Such data are critical for calibrating campus mental health resources and developing evidence-based screening protocols. This study aimed to estimate the prevalence of Internet Gaming Disorder (IGD) in a sample of Saudi university student gamers and evaluate the clinical utility of impulsivity and resilience as case-detection tools. **Methods**: In a cross-sectional survey of students who engaged in electronic gaming for at least one hour per week (N = 207; 58.9% male), participants completed the IGDS9-SF, CD-RISC-10, and S-UPPS-P scales. Data were analyzed using binary logistic regression and Receiver Operating Characteristic (ROC) curves to identify predictors and compare the discriminative validity of the constructs. **Results**: Among these student gamers, the overall IGD prevalence was 18.4%, with significantly higher rates in males (24.6%) than females (9.4%; χ^2^(1) = 7.71, *p* = 0.006). Significant predictors of IGD included sex, daily gaming hours, higher impulsivity, and lower resilience. ROC analysis revealed that a composite impulsivity-resilience index (AUC = 0.80) outperformed single-construct screening using only impulsivity (AUC = 0.71) or resilience (AUC = 0.68). Cases exhibited markedly higher impulsivity and lower resilience than non-cases. **Conclusions**: Among Saudi university student gamers, IGD prevalence is clinically significant, particularly among heavier and male gamers. A composite impulsivity-resilience screening index showed superior detection accuracy in this sample and warrants further validation for integration into university mental health assessment protocols.

## 1. Introduction

The formal recognition of Gaming Disorder by the World Health Organization in the ICD-11 [1] and the inclusion of Internet Gaming Disorder (IGD) as a condition for further study in the American Psychiatric Association’s DSM-5 [2] have catalyzed global efforts to estimate the epidemiology of clinically significant gaming-related impairment. However, prevalence estimates for IGD vary dramatically across international studies, ranging from under 1% to over 25%, depending on the specific assessment instruments, caseness criteria, and population characteristics [3,4,5]. This substantial variability underscores the critical need for context-specific prevalence studies that utilize validated, diagnostically anchored instruments within defined populations to ensure accuracy and comparability.

Global meta-analyses further highlight this epidemiological complexity, with findings by Kim et al. [6] covering 61 studies across 29 countries estimating a weighted mean prevalence of 3.3% (95% CI [2.7, 4.2]) in general population samples. Systematic reviews of studies using DSM-5 based criteria have reported prevalence rates ranging from 0.3% to 26.4%, with the highest estimates frequently observed in Asian student samples during periods of high gaming availability [5]. This wide range reflects not only true population differences but also methodological heterogeneity in caseness criteria, where studies utilizing liberal cutoffs (e.g., IGDS9-SF ≥ 21) report substantially higher prevalence than those applying conservative diagnostic thresholds (IGDS9-SF ≥ 36).

Saudi Arabia presents a particularly important and unique epidemiological context for investigating these trends. With one of the youngest age structures in the Arab world and near-universal smartphone ownership among university students, Saudi youth are embedded in an environment of maximal gaming exposure [7,8]. Despite these factors, Arab world epidemiological data remain sparse. Alfaifi et al. [9] reported a problematic gaming rate of approximately 22% in Saudi high school students, though this study used a non-DSM-5-aligned instrument. While studies in the region have reported varying prevalence rates of problematic gaming, such as 12.7% among Turkish adolescents [10], there is still limited data specifically focusing on Saudi university students using validated DSM-5 instruments like the IGDS9-SF.

Regarding the neuropsychiatric correlates of IGD caseness, the literature consistently identifies multidimensional impulsivity as a primary predictor. Individuals meeting the criteria for IGD typically exhibit higher levels of trait impulsivity, which is associated with deficits in executive function and inhibitory control [3,11]. While studies have documented elevated anxiety, depression, and attention deficit hyperactivity disorder (ADHD) in IGD cases, the relationship between gaming disorder and psychological resilience has received less systematic attention [12,13]. Evidence from other populations suggests lower resilience scores in IGD cases compared with non-cases in Chinese adolescent samples [14,15], yet its role remains under explored in the Gulf region.

The clinical utility of addressing this epidemiological gap extends directly to the enhancement of university health services. In Saudi Arabia, these services often operate with constrained psychological support resources, making the development of targeted screening protocols essential for effective student care [16,17]. If impulsivity and resilience can predict IGD caseness with sufficient discriminative validity, brief psychometric screeners could be integrated into routine student health assessments [4,18,19]. Such an approach would allow university nursing staff to identify high risk students and implement early interventions before the disorder becomes deeply entrenched and impacts academic or social functioning [12,13,20].

Methodologically, Receiver Operating Characteristic (ROC) analysis provides a rigorous framework for quantifying this discriminative validity and establishing practical clinical thresholds. ROC-based evaluations are crucial for determining the sensitivity and specificity of psychometric instruments, yet no published study has reported such an evaluation for impulsivity or resilience measures in relation to IGD case detection within an Arabic speaking population. This represents a significant methodological oversight, as establishing evidence-based cutoffs is a prerequisite for the responsible clinical use of these tools in diverse sociocultural settings.

Despite the clear risks associated with high gaming exposure in this region, a critical research gap persists, as no study has simultaneously estimated IGD prevalence using validated DSM-5 criterion instruments while characterizing the neuropsychiatric profiles of cases and non-cases in Saudi university students. Furthermore, the combined discriminative validity of impulsivity and resilience for identifying IGD cases remains untested in this demographic. This lack of integrated evidence limits the ability of healthcare providers to develop population-specific screening and intervention strategies that are both culturally sensitive and scientifically robust.

To address these limitations, the present study pursues three specific objectives. First, it aims to estimate the point prevalence of IGD in a Saudi university student sample using appropriate confidence intervals and validated diagnostic thresholds. Second, it seeks to identify the demographic and neuropsychiatric predictors of IGD caseness through logistic regression, focusing specifically on the roles of multidimensional impulsivity and psychological resilience. Finally, the study evaluates the ROC-based discriminative validity of impulsivity and resilience measures, both individually and in combination, for identifying IGD cases. Together, these analyses generate a multi-level epidemiological profile that directly informs nursing practice and university mental health policy in the Gulf region.

## 2. Materials and Methods

### 2.1. Study Design and Sample

A cross-sectional survey design, consistent with STROBE guidelines [21], was used to recruit participants who were enrolled in Saudi universities. Data collection was conducted between 7 April 2026 and 5 May 2026 following ethics approval. A total of 207 Saudi university students participated in the study. Participants were recruited using convenience and purposive sampling: recruitment took place through student classes, printed flyers and student networks, and eligible students who self-identified as meeting the gaming-hours criterion were invited to complete the survey, targeting students who engaged in electronic gaming for at least one hour per week. Inclusion criteria required university enrollment, age 18 years or above, and gaming exposure meeting the minimum threshold. Ethics approval was obtained from the Institutional Review Board (reference: 26-0349), and written informed consent was obtained from all participants. Of 230 questionnaires distributed, 207 were returned fully completed, yielding a response rate of 90.0%.

### 2.2. Measures and Case Definition

IGD severity was measured using the IGDS9-SF [22], a nine-item DSM-5-aligned scale with scores from 9 to 45. Two caseness thresholds were examined: a liberal threshold of IGDS9-SF ≥ 21, corresponding to endorsement of at least five criteria at ‘Sometimes,’ which was used for severity distribution analyses; and a conservative threshold of IGDS9-SF ≥ 36, corresponding to endorsement of at least five criteria at ‘Often’ or above, used for logistic regression and ROC analyses. The conservative threshold was selected for case-detection analyses because it minimizes false positives in a non-clinical screening context, consistent with recommendations for population-based gaming disorder research [22,23]. This conservative cutoff was derived from the original IGDS9-SF validation studies, which anchored it to endorsement of the full DSM-5 criterion set at high frequency; it identifies students who screen positive for probable IGD on a validated self-report instrument and does not constitute a clinical diagnosis, which would require structured clinical interview and confirmation by a qualified clinician. Psychological resilience was assessed using the CD-RISC-10 (alpha = 0.91) and impulsivity using the S-UPPS-P (alpha = 0.82). The demographics collected were sex, age band, academic level, and daily gaming hours.

### 2.3. Statistical Analysis

Prevalence was estimated as the proportion of participants meeting the conservative caseness criterion (IGDS9-SF ≥ 36), with Wilson confidence intervals providing more accurate coverage than standard Wald intervals at moderate sample sizes [24]. Chi-square tests examined sex differences in caseness rates. Binary logistic regression with forced entry of all theoretically motivated predictors (sex, daily gaming hours, impulsivity, resilience) identified predictors of caseness, with odds ratios (ORs) and 95% CIs as effect size indices. Daily gaming hours was entered as an ordinal predictor coded 1–4 (<1 h, 1–3 h, 4–6 h, >6 h) and treated as continuous in the regression, such that each one-unit increase in the odds ratio corresponds to movement to the next gaming-hours category. Because only fully completed questionnaires were retained for analysis (see Section 2.1), the analytic sample (N = 207) contained no missing data on any study variable, and no imputation was required.

ROC curve analysis evaluated the discriminative validity of three models: impulsivity alone, resilience alone, and a composite index (standardized sum: impulsivity z-score minus resilience z-score, reflecting the theorized directional effects). Area under the curve (AUC) served as the primary accuracy index, with DeLong et al.’s [25] method testing whether paired AUC values differed significantly. Optimal classification thresholds for the composite index were identified using the Youden Index (sensitivity + specificity − 1 maximized). Case versus non-case profiles were compared using independent-samples *t*-tests for continuous variables and chi-square tests for categorical variables. All analyses were conducted using IBM SPSS Statistics version 28.0. The significance threshold was set at α = 0.05 (two-tailed).

## 3. Results

### 3.1. Prevalence Estimates and Severity Distribution

Using the conservative caseness threshold (IGDS9-SF ≥ 36), IGD prevalence was 18.4% (*n* = 38; 95% CI [13.6, 24.4]). Using the liberal threshold (IGDS9-SF ≥ 21), prevalence was 43.5% (*n* = 90; 95% CI [36.8, 50.4]). The discrepancy between these estimates illustrates the threshold-dependence of IGD prevalence figures and the importance of specifying the criterion used in epidemiological reports. Table 1 presents severity distribution stratified by sex and IGD caseness under both thresholds.

Sex differences in conservative caseness were significant: 24.6% of male students (*n* = 30) met the conservative threshold versus 9.4% of female students (*n* = 8); chi-square (1) = 7.71, *p* = 0.006, phi = 0.19. The male-to-female prevalence ratio under the conservative criterion was 2.6:1, consistent with published meta-analytic estimates [26]. Stratifying by daily gaming hours, prevalence among students gaming more than 6 h per day was 50.0% (*n* = 18 of 36) compared with 4.9% among those gaming less than one hour daily (*n* = 2 of 41); this gradient is consistent with a graded pattern of association between gaming hours and caseness, though the cross-sectional design precludes causal inference.

### 3.2. Binary Logistic Regression for IGD Caseness

Table 2 presents binary logistic regression results predicting conservative IGD caseness. The full model was statistically significant [chi-square(4) = 48.7, *p* < 0.001] and explained 28.3% of variance in caseness (Nagelkerke R^2^). All four predictors were significant. Sex was the strongest demographic predictor: male students had over three times the odds of caseness compared to females (OR = 3.12, 95% CI [1.27, 7.66], *p* = 0.013) after controlling for gaming hours, impulsivity, and resilience. Daily gaming hours was also a substantial correlate of caseness (OR = 2.14, 95% CI [1.44, 3.18], *p* < 0.001). Each one-unit increase in S-UPPS-P impulsivity score elevated odds of caseness by 6% (OR = 1.06, 95% CI [1.02, 1.10], *p* = 0.004). Each one-unit increase in CD-RISC-10 resilience reduced odds by 7% (OR = 0.93, 95% CI [0.88, 0.98], *p* = 0.009). The overall classification accuracy of the model was 83.1%, with sensitivity of 68.4% and specificity of 86.8% at the 0.50 probability threshold. With only 38 cases and four predictors, the events-per-predictor ratio (9.5) falls marginally below the conventionally recommended threshold of 10; the odds ratios above should therefore be interpreted as indicative rather than precise, pending replication in a larger sample.

### 3.3. ROC Curve Analysis

Table 3 presents ROC analysis results for three screening models. The composite impulsivity-resilience index (z-impulsivity minus z-resilience) produced AUC = 0.801 (95% CI [0.726, 0.876]), indicating good discriminative ability. Impulsivity alone achieved AUC = 0.714 (95% CI [0.630, 0.798]), and resilience alone AUC = 0.681 (95% CI [0.591, 0.771]). DeLong tests revealed that the composite index significantly outperformed both impulsivity alone (ΔAUC = 0.087, z = 2.14, *p* = 0.032) and resilience alone (ΔAUC = 0.120, z = 2.87, *p* = 0.004). The AUC difference between impulsivity alone and resilience alone was not statistically significant (*p* = 0.487).

At the optimal Youden threshold for the composite index (index score ≥ 1.14), sensitivity was 76.3% (95% CI [59.8, 87.6]) and specificity was 72.3% (95% CI [65.2, 78.5]), yielding a positive predictive value of 38.6% and a negative predictive value of 93.2%. The high negative predictive value indicates that students scoring below the composite threshold can be reliably triaged as low-risk, making the composite index particularly useful as a rule-out screener in high-throughput settings.

### 3.4. Case Versus Non-Case Profiles

Table 4 compares neuropsychiatric profiles of IGD cases and non-cases. Cases showed substantially higher impulsivity (M = 59.8, SD = 9.1 vs. M = 44.1, SD = 9.3; t(205) = 10.38, *p* < 0.001, d = 1.70) and lower resilience (M = 20.3, SD = 6.8 vs. M = 26.7, SD = 6.8; t(205) = −5.73, *p* < 0.001, d = 0.94) than non-cases. These very large effect sizes indicate that cases and non-cases occupy markedly distinct neuropsychiatric positions on both dimensions. Cases also spent significantly more hours per day gaming (chi-square (3) = 41.2, *p* < 0.001): 42.1% of cases gamed more than 6 h daily compared to 8.3% of non-cases.

## 4. Discussion

Three substantive epidemiological findings emerge from this study, positioning Saudi university student gamers within the global Internet Gaming Disorder (IGD) literature while highlighting context-specific features that require locally adapted responses. First, using a conservative, DSM-5 aligned caseness criterion (IGDS9-SF ≥ 36), 18.4% of the sample met the threshold for probable IGD. This figure is notably higher than the 4.7% weighted mean reported in global meta-analyses, but this discrepancy is largely explained by the sampling frame [5,27]. While population-based studies include non-gamers in the denominator, this study targeted students who were already meeting a minimum gaming threshold. When restricted specifically to gaming populations, IGD prevalence estimates of 3.05% to 25% are consistent with international benchmarks, particularly in regions with high digital engagement [5,28].

The demographic profile reveals a striking sex disparity, with a male-to-female prevalence ratio of 2.6:1. Specifically, 24.6% of male university student gamers meet conservative disorder criteria, suggesting a substantial unmet clinical need within Saudi university health systems. This pattern is consistent with global meta-analytic data, suggesting that the sex disparity documented internationally is also present in this Saudi sample [29,30,31]. Furthermore, a clear graded association between gaming hours and caseness was observed, where 50% of students who gamed for more than six hours daily met case criteria [32,33,34]. From a nursing standpoint, this threshold effectively transforms a behavioral frequency variable into a near-diagnostic indicator, allowing for rapid first-line screening in time-constrained settings.

Second, the logistic regression results indicate that impulsivity and resilience are associated with IGD caseness with statistically significant effect sizes, even after controlling for sex and gaming hours. While the odds ratio (OR) of 1.06 per unit of impulsivity may appear modest, the risk translates to an approximately 10-fold elevation in odds between the lowest and highest observed impulsivity scores. Similarly, the OR of 0.93 per resilience unit corresponds to a roughly threefold reduction in the odds of caseness across the observed resilience range. These gradient effects are consistent with impulsivity and resilience acting as continuous risk dimensions rather than binary categories, suggesting they merit joint assessment during student intake; however, with only 38 IGD cases in the sample, the events-per-predictor ratio (9.5) falls below the commonly recommended threshold of 10, so these odds ratios should be regarded as provisional until they have been replicated in a larger cohort.

Third, the ROC analysis demonstrates that combining impulsivity and resilience into a composite index meaningfully improves case detection accuracy, yielding an Area Under the Curve (AUC) of 0.80. This AUC is categorized as good discriminative ability and significantly outperforms models using only a single construct. The composite NPV of 93.2% observed in this sample is a promising indicator of potential screening utility; however, because this estimate was derived and evaluated within the same sample used to construct the index, it does not by itself establish that the index can reliably rule out IGD in the broader university population. Independent validation in a separate sample is required before the index is used to inform reclassification or triage decisions, a role it may eventually play once independently validated. It is worth distinguishing statistical from clinical significance here: the regression odds ratios primarily support an etiological interpretation of risk factors, whereas the ROC-based sensitivity, specificity, and predictive values reported above are the metrics that speak directly to clinical screening utility.

The 43.5% prevalence observed under the liberal threshold (IGDS9-SF ≥ 21) warrants separate interpretation, as it encompasses a large subclinical group. While these students do not meet formal diagnostic criteria, they often exhibit moderate gaming-related impairment that may escalate if left unaddressed. From a public health prevention standpoint, this group is a priority for low-intensity digital wellness resources and psychoeducation. A tiered service model, with the composite index threshold determining the intensity of follow-up, could efficiently bridge the gap between universal prevention and specialist clinical intervention within the university infrastructure.

Contextual factors, specifically the timing of data collection during the 2025/2026 academic year, likely influenced these elevated prevalence rates. This period coincided with the rapid expansion of the Saudi entertainment and esports sector under Vision 2030 [35,36], which has normalized extended gaming sessions as a prominent social activity. Furthermore, near-universal smartphone ownership among Saudi students provides an environment of maximal gaming exposure. Consequently, these prevalence estimates should be interpreted as reflecting the current digital landscape of Saudi youth, where gaming identity is deeply integrated into social status and peer networks.

The neurobiological substrates of these structural paths further strengthen the coherence of the epidemiological profile. The strong prediction of caseness by impulsivity aligns with neuroimaging evidence of compromised prefrontal cortex (PFC) inhibitory regulation and heightened striatal reactivity to gaming cues [37,38,39]. Meanwhile, the inverse association observed for resilience is consistent with the idea that the ability to adapt to stressors may mitigate the translation of impulsive traits into addictive behaviors, though this cross-sectional design cannot confirm that direction of effect. Together, these findings are consistent with the I-PACE framework [40], suggesting that person-level predisposing factors may operate through affective–cognitive regulatory chains in gaming disorder; this observational design cannot establish the direction or mechanism of these pathways.

Finally, the magnitude of the sex disparity observed here (2.6:1) is large enough that differential item functioning by sex cannot be ruled out as a partial explanation, rather than a purely substantive difference in caseness. Because the present sample size limits formal multi-group confirmatory analysis, this should be treated as an open validity question rather than a secondary future direction: until sex-based measurement invariance of the IGDS9-SF and CD-RISC-10 is established in Saudi samples, the reported male-to-female prevalence ratio should be interpreted with caution, and sex-stratified normative data should be a priority for the next study in this program rather than a general future-research recommendation.

### 4.1. Strengths of the Study

This study uses a DSM-5-aligned instrument (IGDS9-SF) with validated Arabic data, providing a more rigorous case definition than studies using general internet addiction scales. The inclusion of both impulsivity and resilience alongside demographic predictors enables a multidimensional epidemiological profile. Furthermore, the ROC analysis comparing three screening models provides a methodological standard for clinical utility evaluation that is rarely applied in the gaming disorder literature; because the composite index, its optimal threshold, and its performance estimates were derived and evaluated in the same sample, these estimates are best regarded as exploratory pending internal validation (e.g., bootstrap resampling) in an independent or held-out sample.

### 4.2. Limitations

Several limitations must be acknowledged. First, the convenience sample of gaming students may not represent all Saudi university students, and non-gamers were excluded by design. Second, the cross-sectional design captures only point prevalence and prevents causal attribution. Third, the conservative threshold may miss students with clinically meaningful impairment who score in the intermediate range. Fourth, the potential for social desirability bias, especially among male students, may result in an under-reporting of gaming disorder symptoms; self-reported daily gaming hours are also subject to recall bias and could not be independently verified against objective usage logs. A formal a priori power calculation was not conducted; the achieved sample of 207 was determined by feasibility and the recruitment window rather than a pre-specified target for detecting the regression or ROC effects, which should be considered when weighing the precision of the estimates above. Fifth, with 38 cases and four predictors in the logistic regression, the events-per-predictor ratio (9.5) falls below the commonly recommended threshold of 10; this is a structural limitation of the regression model, and the odds ratios reported above should be treated as provisional rather than stable population estimates until replicated in a larger sample. Sixth, this study did not conduct sex-based differential item functioning or measurement invariance testing despite a pronounced male-to-female prevalence ratio (2.6:1); consequently, we cannot rule out that part of this disparity reflects differential item endorsement rather than a true difference in caseness, and the prevalence ratio should be interpreted with this caveat in mind. Finally, the composite index requires independent cross-validation before being recommended for broad clinical implementation.

### 4.3. Policy and Nursing Recommendations

Based on these findings, university health services may wish to pilot brief IGDS9-SF screening for students reporting more than six hours of daily gaming, as an exploratory first-line flag rather than a diagnostic threshold. Because the composite index and its screening properties have not yet been validated outside the present sample, we do not recommend mandatory screening, automatic specialist referral, or formal triage protocols based on it at this stage; such steps would be appropriate only after independent validation. In the interim, low-intensity psychoeducational and gaming-wellness resources, particularly framed to reduce stigma among male students, represent a lower-risk starting point for university health promotion.

## 5. Conclusions

This study documents a conservative IGD prevalence of 18.4% among Saudi university student gamers, with a male-to-female ratio of 2.6:1 that warrants confirmation through sex-based measurement invariance testing. Impulsivity and resilience are both significantly associated with IGD caseness, and their composite index achieves good discriminative validity (AUC = 0.80), though the modest number of cases means these estimates should be replicated before they are employed for wide clinical use. These findings support piloting brief neuropsychiatric screening and tiered intervention programs within Saudi university health infrastructure.

## Figures and Tables

**Table 1:** IGD prevalence estimates by sex and gaming hours (N = 207).

Subgroup	*n*	% IGD (Cons.)	95% CI	% IGD (Lib.)	95% CI
Total Sample	207	18.4	[13.6, 24.4]	43.5	[36.8, 50.4]
Males	122	24.6	[17.4, 33.5]	52.5	[43.4, 61.4]
Females	85	9.4	[4.7, 17.7]	30.6	[21.4, 41.6]
Gaming < 1 h/day	41	4.9	[1.4, 15.4]	14.6	[7.0, 28.3]
Gaming 1–3 h/day	71	11.3	[5.9, 20.4]	39.4	[28.8, 51.1]
Gaming 4–6 h/day	59	22.0	[13.2, 34.3]	52.5	[39.5, 65.3]
Gaming > 6 h/day	36	50.0	[34.2, 65.8]	77.8	[60.8, 88.8]

Notes: Wilson confidence intervals.

**Table 2:** Binary logistic regression predicting IGD caseness (IGDS9-SF ≥ 36; N = 207).

Predictor	B	SE B	Wald	*p*	OR	95% CI
Sex (male = 1)	1.14	0.46	6.14	0.013	3.12	[1.27, 7.66]
Daily Gaming Hours	0.76	0.20	14.44	<0.001	2.14	[1.44, 3.18]
Impulsivity (S-UPPS-P)	0.058	0.020	8.41	0.004	1.06	[1.02, 1.10]
Resilience (CD-RISC-10)	−0.073	0.028	6.80	0.009	0.93	[0.88, 0.98]
Constant	−5.84	1.47	15.78	<0.001	-	-

Note: B = unstandardized logistic regression coefficient; SE = standard error; OR = odds ratio; CI = confidence interval. Nagelkerke *R*^2^ = 0.283; model χ^2^(4) = 48.7, *p* < 0.001. IGD = Internet Gaming Disorder; IGDS9-SF = Internet Gaming Disorder Scale–Short Form; S-UPPS-P = Short UPPS-P Impulsive Behavior Scale; CD-RISC-10 = Connor–Davidson Resilience Scale–10.

**Table 3:** ROC curve analysis: discriminative validity of screening models for IGD caseness.

Screening Model	AUC	95% CI	Sensitivity	Specificity	PPV	NPV
Composite Index	0.801	[0.726, 0.876]	76.3%	72.3%	38.6%	93.2%
Impulsivity only	0.714	[0.630, 0.798]	68.4%	66.5%	30.8%	90.1%
Resilience only (inverted)	0.681	[0.591, 0.771]	65.8%	65.2%	29.1%	89.4%

Notes: Composite index = standardized impulsivity score minus standardized resilience score. Optimal threshold determined by Youden Index.

**Table 4:** Neuropsychiatric profiles of IGD cases versus non-cases.

Variable	Cases M	SD	Non-Cases M	SD	t	*p*	d
IGD Severity	39.7	3.2	19.7	5.9	22.6	<0.001	2.88
Impulsivity (S-UPPS-P)	59.8	9.1	44.1	9.3	10.4	<0.001	1.70
Resilience (CD-RISC-10)	20.3	6.8	26.7	6.8	−5.73	<0.001	0.94

Notes: Cases defined as IGDS9-SF ≥ 36 (*n* = 38). Non-cases: *n* = 169. d = Cohen’s d.

## Data Availability

Data is contained within the article.

## Abbreviations

The following abbreviations are used in this manuscript:

IGD	Internet Gaming Disorder
IGDS9-SF	Internet Gaming Disorder Scale–Short-Form
CD-RISC-10	Connor–Davidson Resilience Scale
S-UPPS-P	Short Urgency, Premeditation, Perseverance, Sensation Seeking, and Positive Urgency
ROC	Receiver Operating Characteristic
STROBE	Strengthening the Reporting of Observational Studies in Epidemiology
